# Supplementary material for: The nematode worm C. elegans chooses between bacterial foods as if maximizing economic utility
Source: eLife. 2023 Apr 25;12:e69779. doi: 10.7554/eLife.69779 (PMC10231927; doi:10.7554/eLife.69779)
Supplement: Supplementary file 1. — Boxes are groups of choice sets in which the price of one food was constant (shading) while the other was variable. Numbers are optical density from which price was computed by Equation 2. Lower case letters refer to data points in Figure 5B. [file elife-69779-supp1.docx]

|  | ***d*** | ***e*** | ***g*** |
| --- | --- | --- | --- |
| **H** | 1 | 1 | 1 |
| **M** | 3 | 1 | 0.1 |
|  |  |  |  |
|  | ***a*** | ***b*** | ***d*** |
| **H** | 0.015 | 0.1 | 1 |
| **M** | 3 | 3 | 3 |
|  |  |  |  |
|  | ***c*** | ***e*** | ***f*** |
| **H** | 0.1 | 1 | 3 |
| **M** | 1 | 1 | 1 |

**Supplementary File 1: Design of the analysis in Figure 6A.** Boxes are groups of choice sets in which the price of one food was constant (*shading*) while the other was variable. Numbers are optical density from which price was computed by equation 2. Lower case letters refer to data points in Figure 5B.
