# Supplementary material for: The nematode worm C. elegans chooses between bacterial foods as if maximizing economic utility
Source: eLife. 2023 Apr 25;12:e69779. doi: 10.7554/eLife.69779 (PMC10231927; doi:10.7554/eLife.69779)
Supplement: Supplementary file 2. — These equations show how the economic variables in the left column were computed based on food type, density and preference in trained and untrained animals. The quantities wT and wU are defined in Equation 15 in the main text. The quantities UT and UU are, respectively, utility in trained and untrained animals computed according to the CES function as fitted to the data in Figure 6C and D. [file elife-69779-supp2.docx]

|  | **Trained** | **Untrained** |
| --- | --- | --- |
| **Offer value H** | $w_{T}{\cdot d}_{H}$ | $w_{U}{\cdot d}_{H}$ |
| **Log offer value H** | $log( w_{T}\cdot d_{H})$ | $log(w_{U}\cdot d_{H})$ |
| **Offer value M** | $d_{M}$ | $d_{M}$ |
| **Log offer value M** | $\log\left( d_{M} \right)$ | $\log\left( d_{M} \right)$ |
| **Offer value** | $w_{T}{\cdot d}_{H}+ d_{M}$ | $w_{U}{\cdot d}_{H}+ d_{M}$ |
| **Log offer value** | $log(w_{T}{\cdot d}_{H}+ d_{M})$ | $log(w_{U}{\cdot d}_{H}+ d_{M})$ |
| **Δ Offer value** | $-w_{T}{\cdot d}_{H}+ d_{M}$ | $-w_{U}{\cdot d}_{H}+ d_{M}$ |
| **Δ Log offer value** | $-log(w_{T}{\cdot d}_{H})+log(d_{M})$ | $-log(w_{U}{\cdot d}_{H})+log(d_{M})$ |
| **Chosen value** | $w_{T}{\cdot f}_{H}{\cdot d}_{H}+(1-f_{H}){\cdot d}_{M}$ | $w_{U}{\cdot f}_{H}{\cdot d}_{H}+(1-f_{H}){\cdot d}_{M}$ |
| **Log chosen value** | $log(w_{T}{\cdot f}_{H}{\cdot d}_{H}+(1-f_{H}){\cdot d}_{M})$ | $log(w_{U}{\cdot f}_{H}{\cdot d}_{H}+(1-f_{H}){\cdot d}_{M})$ |
| **Δ Chosen value** | $-w_{T}{\cdot f}_{H}{\cdot d}_{H}+(1-f_{H}){\cdot d}_{M}$ | $-w_{U}{\cdot f}_{H}{\cdot d}_{H}+(1-f_{H}){\cdot d}_{M}$ |
| **Δ Log chosen value** | $-\log\left( w_{T}{\cdot f}_{H}{\cdot d}_{H} \right)+\log\left( \left( 1-f_{H} \right){\cdot d}_{M} \right)$ | $-\log\left( w_{U}{\cdot f}_{H}{\cdot d}_{H} \right)+\log\left( \left( 1-f_{H} \right){\cdot d}_{M} \right)$ |
| **Offer utility H** | $U_{T}(d_{H},0)$ | $U_{U}(d_{H},0)$ |
| **Offer utility M** | $U_{T}(0, d_{M})$ | $U_{U}(0, d_{M})$ |
| **Offer utility** | $U_{T}(d_{H}, d_{M})$ | $U_{U}(d_{H}, d_{M})$ |
| **Δ Offer utility** | ${-U}_{T}\left( d_{H}, 0 \right)+U_{T}(0, d_{M})$ | ${-U}_{U}\left( d_{H}, 0 \right)+U_{U}(0, d_{M})$ |
| **Chosen utility** | $U_{T}(f_{H}{\cdot d}_{H}, (1-f_{H}){\cdot d}_{M})$ | $U_{U}(f_{H}{\cdot d}_{H}, (1-f_{H}){\cdot d}_{M})$ |
| **Δ Chosen utility** | ${-U}_{T}\left( f_{H}{\cdot d}_{H}, 0 \right)+U_{T}(0, (1-f_{H}){\cdot d}_{M})$ | ${-U}_{U}\left( f_{H}{\cdot d}_{H}, 0 \right)+U_{U}(0, (1-f_{H}){\cdot d}_{M})$ |

**Supplementary File 2: Linear correlation equations**

These equations show how the economic variables in the left column were computed based on food type, density and preference in trained and untrained animals. The quantities $w_{T}$ and $w_{U}$ are defined in equation 15 in the main text. The quantities $U_{T}$ and $U_{U}$ are, respectively, utility in trained and untrained animals computed according to the CES function as fitted to the data in Figure 6C,D.
