# Supplementary material for: The nematode worm C. elegans chooses between bacterial foods as if maximizing economic utility
Source: eLife. 2023 Apr 25;12:e69779. doi: 10.7554/eLife.69779 (PMC10231927; doi:10.7554/eLife.69779)
Supplement: Supplementary file 3. — Significance of correlations was tested using the F distribution. This table shows, for each economic variable tested, the correlation coefficient, value of the F statistic, its two degrees of freedom, and the corresponding p-value. No significant correlations where found. For definitions of variables see Supplementary file 2. [file elife-69779-supp3.docx]

|  | **Trained** | | | | | **Untrained** | | | | |
| --- | --- | --- | --- | --- | --- | --- | --- | --- | --- | --- |
|  | *r*^2^ | *F* | DF1 | DF2 | *P* | *r*^2^ | *F* | DF1 | DF2 | *P* |
| **Offer value H** | 0.004 | 0.021 | 1 | 5 | 0.891 | 0.017 | 0.086 | 1 | 5 | 0.781 |
| **Log offer value H** | 0.255 | 1.710 | 1 | 5 | 0.248 | 0.134 | 0.774 | 1 | 5 | 0.419 |
| **Offer value M** | 0.106 | 0.594 | 1 | 5 | 0.476 | 0.005 | 0.027 | 1 | 5 | 0.875 |
| **Log offer value M** | 0.017 | 0.087 | 1 | 5 | 0.780 | 0.010 | 0.049 | 1 | 5 | 0.834 |
| **Offer value** | 0.003 | 0.014 | 1 | 5 | 0.912 | 0.015 | 0.077 | 1 | 5 | 0.793 |
| **Log offer value** | 0.174 | 1.053 | 1 | 5 | 0.352 | 0.046 | 0.240 | 1 | 5 | 0.645 |
| **Δ Offer value** | 0.006 | 0.029 | 1 | 5 | 0.872 | 0.017 | 0.089 | 1 | 5 | 0.778 |
| **Δ Log offer value** | 0.174 | 1.053 | 1 | 5 | 0.352 | 0.046 | 0.240 | 1 | 5 | 0.645 |
| **Chosen value** | 0.001 | 0.003 | 1 | 5 | 0.959 | 0.000 | 0.002 | 1 | 5 | 0.968 |
| **Log chosen value** | 0.098 | 0.541 | 1 | 5 | 0.495 | 0.007 | 0.035 | 1 | 5 | 0.858 |
| **Δ Chosen value** | 0.004 | 0.019 | 1 | 5 | 0.896 | 0.013 | 0.068 | 1 | 5 | 0.805 |
| **Δ Log chosen value** | 0.199 | 1.241 | 1 | 5 | 0.316 | 0.059 | 0.314 | 1 | 5 | 0.600 |
| **Offer utility H** | 0.162 | 0.965 | 1 | 5 | 0.371 | 0.098 | 0.541 | 1 | 5 | 0.495 |
| **Offer utility M** | 0.035 | 0.183 | 1 | 5 | 0.687 | 0.003 | 0.016 | 1 | 5 | 0.903 |
| **Offer utility** | 0.139 | 0.804 | 1 | 5 | 0.411 | 0.147 | 0.865 | 1 | 5 | 0.395 |
| **Δ Offer utility** | 0.151 | 0.890 | 1 | 5 | 0.389 | 0.043 | 0.225 | 1 | 5 | 0.655 |
| **Chosen utility** | 0.125 | 0.715 | 1 | 5 | 0.436 | 0.130 | 0.749 | 1 | 5 | 0.426 |
| **Δ Chosen utility** | 0.105 | 0.586 | 1 | 5 | 0.478 | 0.130 | 0.749 | 1 | 5 | 0.426 |

**Supplementary File 3: Tests of linear correlations between AWC activation and economic variable it might hypothetically represent**

Significance of correlations was tested using the *F* distribution. This table shows, for each economic variable tested, the correlation coefficient, value of the *F* statistic, its two degrees of freedom, and the corresponding *P* value. No significant correlations where found. For definitions of variables see Supplementary File 2.
